# Historical dimensions of population structure in a continuously distributed marine species: The case of the endemic Chilean dolphin

**DOI:** 10.1038/srep35507

**Published:** 2016-10-19

**Authors:** M. J. Pérez-Alvarez, C. Olavarría, R. Moraga, C. S. Baker, R. M. Hamner, E. Poulin

**Affiliations:** 1 Instituto de Ecología y Biodiversidad (IEB), Facultad de Ciencias, Universidad de Chile, Las Palmeras 3425, Ñuñoa, Santiago, Chile; 2 Centro de Investigación Eutropia, Ahumada 131 Oficina 912, Santiago, Chile; 3 Fundación CEQUA, 21 de Mayo 1690, Punta Arenas, Chile; 4 Centro de Estudios Avanzados en Zonas Áridas (CEAZA), Raúl Bitrán1305, La Serena, Chile; 5 Marine Mammal Institute and Department of Fisheries and Wildlife, Oregon State University, Hatfield Marine Science Center, 2030 SE Marine Science Drive, Newport, OR 97365, USA

## Abstract

The complementarity of historical and contemporary processes contributes to understanding the genetic structure of continuously distributed marine species with high dispersal capabilities. *Cephalorhynchus eutropia,* has a continuous coastal distribution with strong genetic differentiation identified by nuclear DNA markers. We explored the historical dimension of this genetic differentiation between northern and southern populations to evaluate phylogeographic structure. Additionally, we conducted mtDNA and microsatellite analyses to detect past and recent demographic changes. The southern population was characterized by lower genetic diversity with a signal of population expansion, likely associated with ice retreat and habitat extension after the Last Glacial Maximum (LGM). In contrast, structure within the northern population was more consistent with stable historical population size. Approximate Bayesian Computation analyses suggested that during the LGM, *C. eutropia* persisted in the northern area; while the south was colonized by dispersal ~11,000 years ago followed by population expansion. This study shows that Chilean dolphin population structure is consistent with predictions from the Expansion-Contraction biogeographic model, with a poleward post-glacial shift revealed in current genetic structure. The results also confirm the validity of the population units previously identified, demonstrating their historical origin and highlighting the utility of integrating genetic markers with different temporal scale resolutions.

Understanding the population structure of marine species represents a challenge as their environments exhibit fewer obvious physical geographic barriers to gene flow than terrestrial ones^1^, and because many species are continuously distributed over apparently homogeneous areas^2^. Moreover, despite a high dispersal potential, some mobile marine species show a high degree of population structuring as described for Atlantic blue fin tuna, *Thunnus thynnus thynnus*^3^, white shark, *Carcharodon carcharias*, in the Mediterranean Sea^4^, and humpback whales, *Megaptera novaeangliae*, in the North Pacific^5^.

Genetic structure in marine populations commonly reflects a historical and contemporary interplay among a combination of ecological, demographic, behavioral, genetic, oceanographic, climatic and other factors^6^. Therefore, an integrative approach for understanding population structure and population boundaries in continuously distributed species is necessary. Population differentiation may be influenced at contemporary time scales by intrinsic factors such as resource specialization^7^, site fidelity to specific feeding and breeding grounds^8^ and social behavior^9^. Also at contemporary time scales, habitat and oceanographic characteristics such as currents, salinity or temperature^10^ may be operating, and *a priori* hypotheses regarding the placement of subpopulation boundaries or patterns of genetic structure may help to explain the observed patterns^7^,^11^. At historical time scales, a concordance between phylogeographic and biogeographic patterns has been widely observed, showing that the forces determining species distributions are also related to the spatial patterns of population genetic structure^12^. In this context, historical processes such as differential effects of glaciation and sea-level changes may greatly influence biogeographic patterns, shaping the distribution and population structure of many species^12^,^13^. As proposed by the Expansion-Contraction (EC) biogeographic model^14^, species directly affected by glacial conditions during Pleistocene climatic cycles would have restricted their range during the coolest periods and then would have re-colonized high latitudes after the ice retreat, which may generate genetic differentiation^15^ and reduced levels of genetic diversity^16^. Most biogeographical studies related to the Pleistocene glacial cycles have been performed in the Northern Hemisphere with only few studies along the coasts of the Southern Hemisphere, where physical, geological and evolutionary context are very different. Glaciations that occurred during the Pleistocene covered a much larger area and had a larger impact in the Northern than in the Southern Hemisphere, primarily due to the disposition of the continents^17^. However, some effects of glaciation on biogeographic patterns and population genetic structure have been inferred from phylogeographic studies in South America including marine taxa such as limpets, bivalves^18^, fishes, *Eleginops maclovinus*^19^, *Galaxias maculatus*^20^ and the southern river otter^21^, among others.

Climatic and oceanographic variations have also contributed substantially to the current distribution pattern, geographic variation and population structure of some species of the Order Cetacea. The population expansion of belugas, *Delphinapterus leucas*, in the western Nearctic occurred after the retreat of the Pleistocene ice cover, and the chronology of postglacial events is thought to have shaped the dispersal routes of this species from its glacial refuges^22^. For white-beaked dolphins (*Lagenorhynchus albirostris*) in the North Atlantic, demographic inference based on mitochondrial DNA suggests a population expansion in the Barents Sea off the Norwegian coast and geographic extension to the British Isles in the North Sea after the Pleistocene glacial events^23^. Associations between distribution and climate changes for harbor porpoise, *Phocoena phocoena*, and minke whale, *Balaenoptera acutorostrata*, in the North Sea were mainly attributed to environmental requirements for feeding and breeding^24^. The abundance and distribution of the gray whale, *Eschrichtius robustus*, in the North Pacific were also modified by variations in sea level and the availability of environments not covered by ice in the late Pleistocene^25^. As illustrated by the examples presented here, also for cetaceans, most of the existing information is derived from studies conducted in the Northern Hemisphere.

The coastal endemic Chilean dolphin (*Cephalorhynchus eutropia*), distributed along the Southeast Pacific coast between 30° and 56°S, represents an interesting model for understanding how a combination of historical and contemporary factors resulted in the current genetic structure of this continuously distributed marine species. Using 21 microsatellite markers, Pérez-Alvarez *et al.*^26^ identified a marked genetic break along its distribution, located between 42° and 46°S, in the absence of obvious geographic barriers separating the two genetically differentiated populations. The differentiation index values for these two populations (F_ST_ = 0.15 and R_ST_ = 0.19) were higher than those reported among populations of most other dolphin species at large^27^ and small geographic scales^28^; these values and the low migration rates estimated^26^ indicate highly restricted gene flow between adjacent populations. In the absence of obvious physical barriers between the northern and southern Chilean dolphin populations, this population differentiation was interpreted as the result of a complex scenario involving local adaptation and behavioral specialization. Differences in oceanographic and topographic characteristics associated with geographic variation of prey items likely contributed to differentiation between these two populations. Additionally, specialized behavior in each area could also be an important factor that reinforces the current genetic structure in Chilean dolphins^26^. Complementary historical factors may also have been involved in generating the observed population genetic structure.

The genetic break between the northern and southern populations coincides with the boundary between two major marine biogeographic units: (1) the northern area is located from 30°S to 42°S (Intermediate Zone or Central/Southern region) characterized by an open and exposed coast with the presence of river runoff, where Chilean dolphins are associated with estuarine habitats^29^,^30^ and (2) the southern area extends from 42°S to 56°S, called the Magellan Province^31^ or the Austral Fjords Region^32^. The southern area is a protected area of fjords and channels dominated by Sub-Antarctic water and the melting of resident glaciers^32^, where Chilean dolphins are mainly located in protected fjords and channels^30^. This biogeographic boundary is also associated with the oceanic divergence between the Humboldt and the Cape Horn currents, where the West Wind Drift reaches the coast and divides into the northward flowing Humboldt Current and the poleward flowing Cape Horn Current. The latter passes between South America and Antarctica through the Drake Passage, and influences both the east and west coasts of South America^33^. Furthermore, the geographic break detected in *C. eutropia* is also coincident with the northern limit of the LGM ice sheet^34^. During this period, western Patagonia was covered by a vast ice sheet that extended from the southernmost latitudes to Chiloé Island, south of 43°S, descending from the Andes and reaching the edge of the continental shelf^35^. Such cold episodes greatly impacted the Patagonian fjords and channels area, directly affecting Chilean dolphin habitat.

Consistent with the basic E-C model, *C. eutropia* may have survived the Last Glacial Maximum (LGM) in low latitude areas, contracting its distribution to areas less impacted by ice, and then recolonizing higher latitudes through range expansion after the ice retreat^14^. Recolonizing populations are usually composed of subsets of the genetic diversity present in the source population and the founding process can result in a series of sequential founder effects and bottlenecks^14^. Alternatively, small populations of Chilean dolphin may have survived throughout the period of glaciation in refugia, from which they recolonized the impacted area during deglaciation^14^. In this study, we used mtDNA analysis to explore the historical dimension of the current genetic structure of the Chilean dolphin, evaluating the existence of phylogeographic structure along its distribution. We also performed demographic inference analyses with both nuclear microsatellites and mtDNA markers to detect signals of past and recent demographic changes. Finally, we analyzed the historical biogeography of *C. eutropia* to test whether population structure as influenced by the LGM is more consistent with the predictions of the expansion-contraction model under the “*In situ* refugia” or “Northern shift” scenarios.

## Results

### Identification of population units

**Figure 1:**
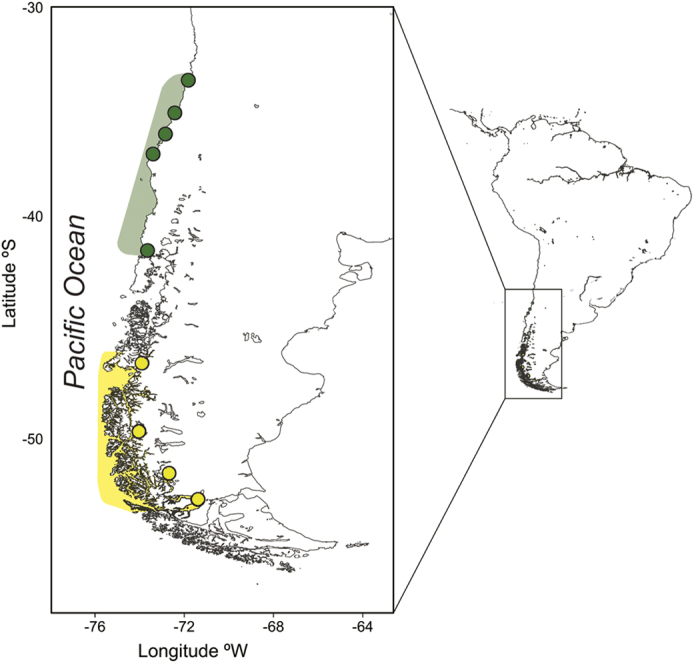
Locations where Chilean dolphins, *Cephalorhynchus eutropia*, were sampled. Circles correspond from north to south to the following localities: San Antonio, Constitución, Melas, Llico, Maullín, Aysén, Bernardo O´Higgings, Puerto Natales, Punta Arenas. Green and yellow areas correspond respectively to Northern and Southern population identified for Chilean dolphin along its complete distribution. Map was designed by corresponding author and plotted by N. Segovia (Acknowledgments section) in R 3.2.2 software https://cran.r-project.org/ using ggplot2 (https://cran.r-project.org/web/packages/ggplot2/citation.html). The source of the base maps was GEODAS, NG, NOAA (https://www.ngdc.noaa.gov/mgg/shorelines/) and Coastline Extractor Software was used (https://www.ngdc.noaa.gov/mgg/geodas/geodas.html).

**Figure 2:**
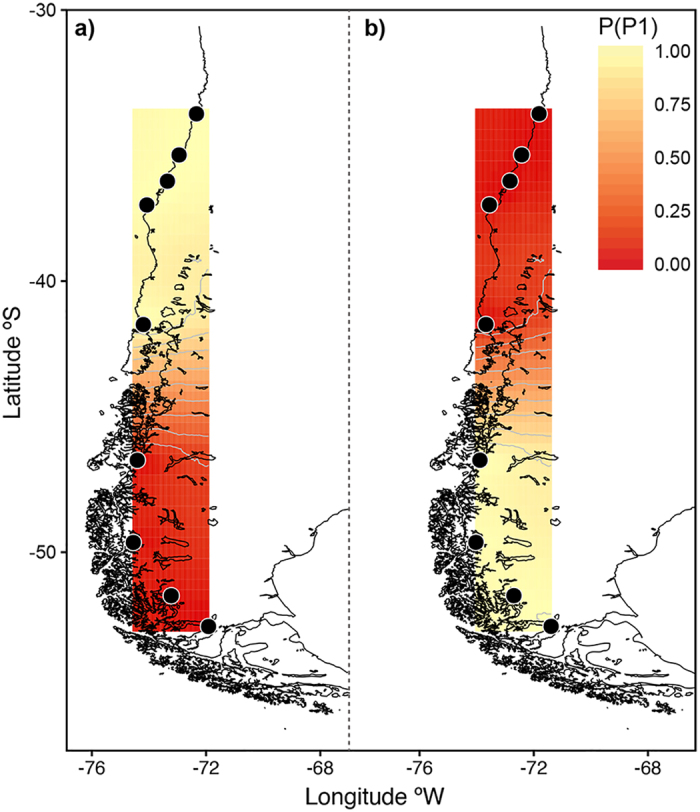
Posterior probabilities of population membership and genetic discontinuities from the spatial model in GENELAND for the Chilean dolphin using mtDNA and microsatellites markers. Contour lines indicate the spatial position of genetic discontinuities and lighter colors indicate higher probabilities of population membership. Two genetic clusters were identified. (**a**) Northern population (**b**) Southern population. The maps were obtained in GENELAND (http://www2.imm.dtu.dk/~gigu/Geneland/) by the corresponding author and plotted by N. Segovia (Acknowledgments section) in R 3.2.2 software (https://cran.r-project.org/) using ggplot2 (https://cran.r-project.org/web/packages/ggplot2/citation.html). The source of the base maps was GEODAS, NG, NOAA (https://www.ngdc.noaa.gov/mgg/shorelines/) and Coastline Extractor Software was used (https://www.ngdc.noaa.gov/mgg/geodas/geodas.html).

Analyzing the microsatellite and mtDNA data simultaneously, the spatial model in GENELAND identified two genetic clusters with a boundary located between Maullín (41°36′S) and Aysén (46.36′S) (Figs 1 and 2). Individuals sampled from the Northern and Southern areas exhibited a high probability of belonging to their respective clusters (P > 0.9).

#### Network

**Figure 3:**
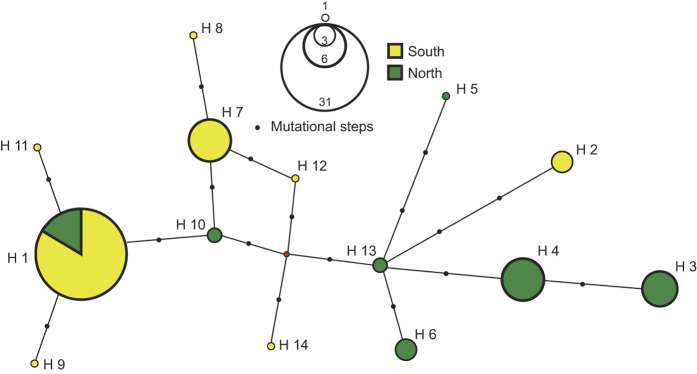
Median-joining network of mtDNA control region haplotypes of the Chilean dolphin. Circle size is proportional to the number of individual sharing a haplotype. Length of the lines is proportional to the number of mutational steps separating haplotypes. Green: Northern area, Yellow: Southern area.

A total of 14 haplotypes were defined by 15 polymorphic sites among the 64 individuals. Overall, mtDNA haplotype diversity (h) was 0.75 ± 0.053 and nucleotide diversity (π) was 0.038 ± 0.002 (Table 1). The Southern population showed less genetic diversity (Northern h = 0.86, π = 0.35%, Southern h = 0.56, π = 0.24%) (Table 2). Relationships among the haplotypes and their relative frequencies are shown in Fig. 3 and Table 1 respectively. The most common haplotype (H1, 29 samples) was the only one shared between the Northern and Southern areas, but at very different frequencies. The Southern population was characterized by the high frequency of haplotype H1 (H1 south = 0.62) while in the northern area, its frequency was much lower (H1 north = 0.23) and comparable to the frequencies of H3 and H4. Most of the other haplotypes were represented by only one or two individuals. Genetic differentiation between two populations was high and significant (Φ_ST_ = 0.39, P < 0.0001 and F_ST_ = 0.19, P < 0.0001). Phylogeographic structure was indicated by the significantly greater value for Φ_ST_ compared to F_ST_ (P = 0.0004).

### Demographic inference

**Figure 4:**
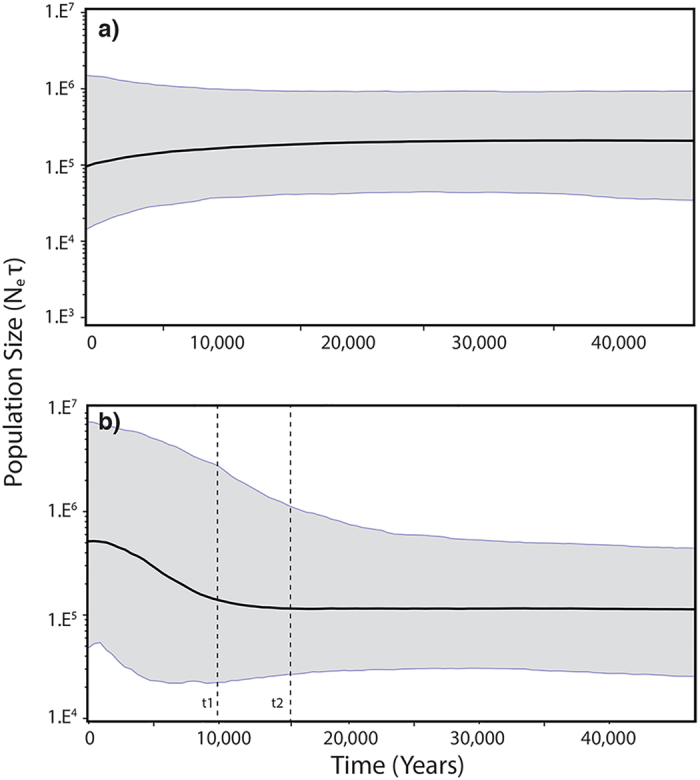
Bayesian skyline plot for the Northern (**a**) and Southern (**b**) populations of the Chilean dolphin. The X axis is in units of years and the Y axis is equal to N_e_τ; (the product of the effective population size and the generation length in years). The time of population expansion (t1) and start time of deglaciation (t2) were obtained by ABC analysis.

Analysis of Bayesian skyline plots of each genetic group showed that the Northern population of Chilean dolphin (Fig. 4a) remained at a stable population size about 14000 from 40,000 to 12,000 years ago and then showed a tendency to decrease, whereas the southern population shows an increase from 8500 to 36000 starting about 11,000 years ago (Fig. 4b). Additionally, for microsatellites, the three statistical tests performed by the Bottleneck program detected significant heterozygosity deficit compared to the heterozygosity expected from the observed allele number at mutation-drift equilibrium. These results were obtained using both the stepwise mutation model (SMM) (Sign test P = 0.01; Standardized differences test P = 0.0006; and Wilcoxon sign-rank test P = 0.01332) and the two-phase model (TPM) (Sign test P = 0.01, standardized differences test P < 0.0001 and Wilcoxon sign-rank test 0.00211). This suggests a past population expansion in the Southern population of Chilean dolphin. In contrast, no significant deviation from the mutation-drift model was detected for the Northern population, and results did not support any recent demographic change in this area.

### Historical scenarios

The ABC (Approximate Bayesian Computation) analysis discriminated between the two proposed scenarios based on geographic population structure and demographic inference of *C. eutropia* along its distribution. The results supported the “Northern shift” scenario with the highest posterior probability 0.81 (CI 95% 0.801–0.825) and 0.99 (CI 95% 0.926–1.000) for mtDNA and microsatellites respectively. Additionally, model checking, direct approach and logistic regression also supported the Northern shift scenario to be the most likely (Supplementary Fig. S1). This suggests that the Southern population of Chilean dolphin would have originated from the Northern population after LGM ice retreat through a postglacial colonization characterized by founder event(s) and rapid population expansion. The type I error rates, i.e. the risk of rejecting the “Northern shift” when it is true, were 0.1% and 1.2% for mtDNA and microsatellites, respectively. The Type II error rates, i.e. the risk of accepting the focal scenario when it is false, were 21% and 2.7% for mtDNA and microsatellites, respectively. These were assessed by the 1,000 nearest simulations. “The higher value for mtDNA Type II error is likely due to the fact that these data represent a single locus, and therefore, a single gene genealogy subject to substantial stochastic variation”^36^. The demographic parameters estimated from the posterior distribution for this scenario are shown in Table 3.

## Discussion

The interpretation of the structure and distribution patterns of genetic diversity is one of the research objectives of fields including molecular ecology, population genetics and landscape genetics^37^. These patterns become more predictable in cases when the genetic structure pattern is concordant with geographic structure^38^ as has been described for example in the mammal *Leopardus guigna* in southern South America, where geographical barriers such as the presence of the Andes Mountains and the Chacao Channel have shaped its current genetic population structure^39^, and in the fish, *Orestias ascotanis*, endemic to the Altiplano, where population structure reflects a long-term consequence of habitat fragmentation^40^.

However, as few absolute barriers to gene flow exist in oceans, even widely separated regions may be genetically connected and many marine species often exhibit low levels of genetic differentiation over large distances^41^. Conversely, despite a high dispersal potential, some marine species show a high degree of population structure^3^,^4^,^5^, and in some circumstances, the interpretation of this genetic structure may be difficult and even speculative. In such cases, it may be useful to explore the historical dimension of the differentiation process by using molecular markers capable of capturing the past signal. In this context, we highlight the effectiveness of integrating genetic markers with different temporal scale resolutions^42^ to evaluate both ongoing microevolutionary processes (*e.g.* using microsatellite loci^42^) and historical biogeography (*e.g.* using mtDNA^42^).

The dolphin genus *Cephalorhynchus* is an example of marine species with widespread but discontinuous distribution in the cool-temperate zone of the Southern Hemisphere^43^. Three of the four species in this genus (Commerson’s dolphin, *C. commersonii;* Hector’s dolphin, *C. hectori*; and Chilean dolphin, *C. eutropia*) have demonstrated genetically divergent and structured populations. In the Commerson’s dolphin, genetic differentiation has been described for populations found along the southeastern coast of South America and around the Kerguelen Islands in the southern Indian Ocean (currently recognized as separate subspecies^44^), where geographical isolation has likely had a strong influence on the genetic structure. On a smaller geographical scale, the Hector’s dolphin shows a strong genetic differentiation between the North and South Island of New Zealand^43^,^45^, which are separated by the Cook Strait. The deep water of Cook Strait is likely acting as a barrier to dispersal and gene flow between the North and South Island populations (currently recognized as separate subspecies^45^). And finally, for the Chilean dolphin, microsatellite data revealed a strong genetic break which divides the species into northern and southern populations within a continuous distribution^26^. In contrast to *C commersonii* and *C. hectori*, the continuous distribution of the Chilean dolphin (*C. eutropia*) and the absence of a geographic gap or barrier separating the genetically distinct populations make its current genetic structure puzzling. Nevertheless, from a historical point of view, the genetic break between Northern and Southern populations appears to coincide with the boundary between two major marine biogeographic units^31^,^32^ and with the northern limit of the ice sheet along the Southeast Pacific coast during the LGM^34^. These factors were the most likely influences shaping the genetic structure of this dolphin species. Climatic change and specifically glacial cycles are considered one of the main factors influencing biogeographic patterns of distribution and population structure for many species at different temporal and spatial scales^13^. Biogeographic studies related to glacial cycles provided the empirical basis for the Expansion-Contraction (E-C) model of Pleistocene biogeography^14^. According to this model, species directly affected by glacial conditions during Pleistocene climatic cycles would have restricted their range during the coolest periods and then re-colonized high latitudes after the ice retreat, and that may have generated genetic differentiation^15^ and reduced levels of genetic diversity^16^. Following this model, predictions can be made about the contrasting levels and patterns of genetic diversity that should be detected between populations sampled in ice-affected *vs.* non-affected areas during glacial cycles.

In this context, the present study shows that the predictions of the Expansion-Contraction model are consistent with the population structure, genetic diversity and demographic history of the Chilean dolphin:

1. A signal of population structure is predicted, as recolonization from non-affected areas will likely produce genetic differentiation between areas affected and others not affected by the glacial cycles due to genetic drift or alternatively, long-term isolation of populations that persisted within *in situ* refugia^14^. In this context, two latitudinally divided genetic population units (Northern and Southern Chilean dolphin populations) have been identified. These populations are separated by a strong genetic break as indicated by a high population differentiation index for both microsatellites (F_ST_ = 0.15 and R_ST_ = 0.19, Pérez-Alvarez *et al.*^26^) and the mtDNA control region (Φ_ST_ = 0.39, P < 0.0001 and F_ST_ = 0.19, P < 0.0001). The geographical boundary between these two population units coincides with the extension of the LGM ice sheet^35^, delimiting two populations affected and unaffected by the glacial periods.
2. Less genetic diversity is predicted in areas recolonized postglacially compared to populations that remained in refugia or at lower latitudes as consequences of the ice-free recolonization process, founder effect and genetic drift^14^. The Southern population of Chilean dolphin, located in an area affected by glacial events, shows the lowest genetic diversity (haplotype and nucleotide) values, and a haplotype genealogy characterized by a dominant haplotype, from which low frequency haplotypes were derived.
3. Finally, population growth that follows the recolonization process is predicted to generate a population expansion signal in deglaciated areas. For the Chilean dolphin, Northern and Southern populations exhibit different demographic histories. Demographic inference analyses (based on a coalescent model), revealed a relatively stable population size for the last 40,000 years for the Northern population, after which a non-significant trend of population decrease is observed. This signal could reflect a current decrease in this population maybe related to human activities. In contrast, for the Southern population, Bayesian skyline plots as well as ABC estimated parameters, showed a population size increase from approximately 11,000 years ago. In both cases, coalescent method (Fig. 4) tends to overestimate effective population size compared to ABC approach (Table 3). Population growth was also detected in the Southern area by means of microsatellite markers. In this case, as expected in recently expanding populations, genetic diversity is lower than the expected value under the mutation drift equilibrium simulated from the allele number^46^.

Overall, our results suggest that the southern Chilean dolphin population went through an expansion process after LGM ice retreat by population expansion initiated about 11,000 years ago. This is consistent with the chronological dating information of glacial cycles reported for these latitudes, particularly during the LGM. The time period estimated for the glacial advance to the northern limit of Pacific Patagonia is approximately 17,900 years ago, and the deglaciation would have begun about 17,500 years ago, synchronously throughout Patagonia^47^. Similarly, in the Strait of Magellan, the final glacial advance occurred about 17,000 years ago and the largest and most rapid deglaciation period occurred between 14,000 and 10,000 years ago^35^,^48^. The complete ice retreat around Tierra del Fuego and the Beagle Channel dates from about 11,600 years ago.

The population genetic diversity and structure of the Chilean dolphin can be analyzed relative to two alternative E-C historical scenarios. The “*in situ* refugia” scenario involves the persistence of species in refugia within the glaciated area, followed by expansion within the higher-latitude area from those refugia. The “Northern shift” scenario involves a contraction in population size and displacement toward lower latitudes during periods of cooling, followed by recolonization of southern glaciated zones during postglacial warming from the northern area. Based on geographic population structure and demographic inferences, The Approximate Bayesian Computation approach supported the “Northern shift” as the more probable scenario. This scenario essentially amounts to restriction of the distribution of *C. eutropia* during the LGM to the area north of Chacao Channel. The exclusion of *C. eutropia* from the southern area during the LGM may be explained by decreased primary productivity, prey distribution and abundance decrease within the area affected by the LGM, and simple physical displacement from ice-covered areas. The absence of this species in areas directly affected by the glacial cycles is consistent with what is known about genus *Cephalorhynchus*, which appears to be restricted to particular and limited habitats in the cool-temperate zone^43^, with no records in high ice-associated latitudes.

In contrast to the findings for the Chilean dolphin, a study on another aquatic mammal found in similar latitudes, the southern river otter (*Lontra provocax*)^21^, suggests a persistent demographic-historic model (*i.e.* “*in situ* refugia”) as the most plausible scenario for that species during the LGM. This is mainly based on the absence of a star-shaped haplotype genealogy, no sign of population growth for the area, and high levels of genetic diversity observed in the area affected by the ice. The persistence of the southern river otter in the glaciated area would have been possible given the survival of other aquatic and marine species that serve as its food source and/or flexibility in its diet as an adaptation to the changing environment during the LGM^21^.

In summary, this study contributes to our understanding of the demographic history of a continuously distributed marine species in relation to the effects of historical climate variation, particularly Pleistocene glacial cycles, on its distribution patterns, population structure and genetic diversity. The strong phylogeographic component supports historical influences on the currently-observed genetic break between the Northern and Southern populations of Chilean dolphin^26^. Analyses of both microsatellite and mtDNA markers support the impact of glacial influence on this genetic pattern, and Chilean dolphin population structure being most consistent with the ‘Northern shift’ scenario of the Expansion-Construction biogeographic model. An integrative methodological approach provided a valuable way to assess current population structure and infer its historical origins, suggesting that Southern population originated after the retreat of the ice, probably as a result of postglacial recolonization from populations located farther north. Still needed is a better understanding as to what has maintained such a profound differentiation of nuclear and mtDNA since the post-glacial expansion. Further studies should evaluate aspects such as natal fidelity, ecological specialization and perhaps the results of selection. However, this research represents an important contribution not only to the study of the Chilean dolphin, but also to the historical biogeography of marine mammals in the Southern Hemisphere, particularly species distributed in areas directly affected by glacial cycles.

## Methods

A total of 66 tissue samples were collected at nine localities along the Chilean coast between 35°20′S 72°25′W and 52°40′S 72°30′W (Fig. 1). Samples were obtained from adult dolphins at sea using skin swabbing (n = 10) and biopsy darting (n = 41), and also from dolphins killed incidentally in fisheries (n = 15). Samples were stored in 90% ethanol and DNA was extracted using the salt extraction method.

### Mitochondrial DNA amplification (Dloop)

A 663 bp fragment of mitochondrial DNA control region (Dloop) was amplified for 64 individuals using the primers M13 Dlp1.5 5′-TGTAAAACGACAGCCAGTTCACCCAAAGCTGRARTTCTA-3′ and 8G 5′GGAGTACTATGTCCTGTAACCA^49^. Amplification reactions were performed in a total volume of 25 μl with 5 ul PCR buffer 10X, 2 μl MgCl2 50 mM, 1 μl of each primer, 2 μl dNTP 200 mM, 0.3 μl Taq DNA polymerase (Invitrogen Life Technologies) and 50 ng DNA. The PCR temperature profile was as follows: a preliminary denaturing period of 2 min at 94 °C followed by 30 cycles of denaturation for 30 s at 94 °C, primer annealing for 40s at 56 °C and polymerase extension for 40 s at 72 °C. A final extension period or 10 min at 72 °C was included. Forward and reverse strands were sequenced using an ABI 3730XL Analyzer by Macrogen Inc. (Korea).

Sequences were edited and aligned in PROSEQ 2.91 and the species was confirmed using a BLAST search undertaken in GeneBank and by DNA Surveillance.

### Genotyping

We used genotype data obtained by Pérez-Alvarez, *et al.*^26^ for 53 *C. eutropia* samples and 21 dinucleotide microsatellite loci: Ev1, EV14, EV37, EV94, EV104^50^; KWM12^51^; MK5, MK6; PPH110, PPH130, PPH137, PPH142^52^; GT023, GT211, Gt575^53^; TtruGT51, TtruGT142^54^; Sgui03, Sgui06, Sgui17^55^ and TexVet5.

### Genetic and phylogeographic structure

To explore spatial boundaries we used GENELAND, a Bayesian model that uses genotypes and spatial coordinates of individuals to cluster them into populations at approximately Hardy-Weinberg equilibrium, considering linkage equilibrium between loci. For this analysis, both markers (mtDNA and 21 microsatellites loci) were used simultaneously. An allele frequency uncorrelated model was set, with 1,000,000 MCMC iterations and thinning of 100.

To evaluate the existence of phylogeographic structure, control region sequences were divided into two groups based on results obtained from GENELAND. Genetic diversity at the haplotype (h) and nucleotide (π) levels was estimated for the complete study area and also for the Northern and Southern areas using ARLEQUIN v 3.5. Considering the differences in sample size between areas, values of haplotype richness were also adjusted to sample size using a rarefaction analysis in PAST software. Genealogical relationships of the haplotypes were investigated by constructing a median-joining network in NETWORK 4.5.1.0. Genetic differentiation between Northern and Southern populations was assessed by calculating **Φ**_ST_ (using pairwise difference distance, assuming Jukes Cantor substitution model) and F_ST_ (using haplotype frequencies) in ARLEQUIN v 3.5. Phylogeographic structure was evaluated using PERMUT Software by comparisons between the observed **Φ**_ST_ and F_ST_ values, using 10,000 permutations. Because F_ST_ is a genetic differentiation index between populations based on haplotype frequency and **Φ**_ST_ considers both haplotype frequencies and genetic distance between haplotypes, a higher value of **Φ**_ST_ compared to F_ST_ reveals the existence of a phylogeographic structure.

### Demographic inference

To assess the patterns of demographic history (1) The past population dynamics (demographic history) of each population was reconstructed by a Bayesian Skyline-plot method implemented in BEAST 1.7, which is based on coalescent theory and quantifies the relationship between the genealogy of the sequences and the demographic history of the population^56^. For the data set analysis a strict clock model was computed considering a mutation rate of 1.5%/myr^57^ and a mutation model (HKY) previously estimated using Mr Modeltest v.2.3 8. The convergence of the Markov chain Monte Carlo (MCMC) chains was observed with the program Tracer v 1.5. Additionally (2) we used the program BOTTLENECK^58^ in order to evaluate whether each Chilean dolphin population (Northern and Southern) had undergone recent effective population size changes. This program detects recent demographic variations comparing for each locus, the observed heterozygosity with the heterozygosity expected at mutation-drift equilibrium, simulated from the observed allele number^46^. The stepwise mutation model (SMM) and two-phase model with 90% stepwise mutations (TPM) was assumed and the probability of heterozygosity deficit was calculated by the statistical sign test, a standardized differences test^45^ and a Wilcoxon sign-rank test.

### Evaluating two historical scenarios potentially involved in the current genetic structure of Chilean dolphin

**Figure 5:**
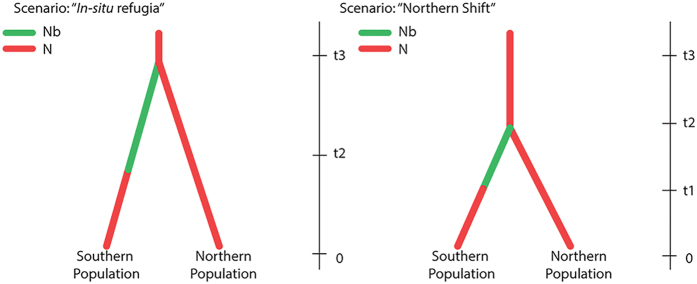
Historical scenarios potentially involved in the current genetic structure of Chilean dolphin. “*In situ* refugia” (left) and “Northern Shift” (right). N: Population size; Nb: Reduced population size (bottleneck or founder effect) t3: Glaciation starting time t2: Deglaciation starting time t1: Population expansion time during ice retreat phase.

To assess the influence of glacial cycles on the demographic history and genetic structure of *C. eutropia*, we used the Approximate Bayesian Computation (ABC) method. This approach makes it possible to test complex population genetic models by simulating the data sets of predefined scenarios and comparing their summary statistics with the summary statistics of the observed data^59^. We compared the posterior probabilities of two competing scenarios of genetic differentiation that are characterized by contrasting population divergence times and demographic histories. The “*in situ* refugia” scenario proposed that the population of *C. eutropia* present in the southern area of distribution (Chacao Channel to the South) experienced reduced population size and survival only in restricted areas during the Last Glacial Maximum (LGM). Subsequently, a population expansion would have occurred in this area after the retreat of the ice. The “Northern shift” scenario considered that during the LGM the distribution of *C. eutropia* was restricted to the area north of Chacao Channel. In this case, the Southern population was originated after the ice retreat through postglacial colonization from the Northern population, and was characterized by founder effects followed by rapid population expansion (Fig. 5). Posterior probabilities were estimated with DIYABC^60^ using historical, demographic and mutational parameters drawn from the prior distributions. For each scenario, 10,000,000 data sets were simulated and the relative likelihoods of scenarios were compared using a logistic regression on 1% of simulated data closest to the observed data set^60^. We used the model-checking function of DIYABC to assess the goodness of fit between each model parameter. The prior distributions for time related parameters (t1: population expansion, t2: deglaciation starting time and t3: glaciation starting time) were set based in historical references of the Last Glacial Maximum (LGM) in South America^34^,^35^,^46^,^48^. Due to the absence of available data for the effective size related parameters (N: Population size and Nb: Reduced population size), or even abundance, we used a uniform and broad priors with the limits exceeding the probable smallest and largest population sizes for this species (Table 3). We assumed a generalized stepwise mutation model with a uniform prior distribution of mean mutation rate from 10^−5^ to 10^−3^, and a prior distribution of individual locus mutation rates from 10^−5^ to 10^−2^ following a Gamma distribution with mean determined by the mean mutation rate across loci. The maximum likelihood of both scenarios was compared by logistic regression^60^ and the posterior probability of fit of each model by computing type I error (risk to reject the focal scenario when it is the true one) and type II error (risk to accept the focal scenario when it is false), which were estimated using the “Confidence in scenario choice” function.

### Approval

All experimental protocols were approved by the Bioethical Committee at the Universidad de Chile and biopsy samples were collected under permit from the Chilean Under Fisheries RES 665/2009, RES 67/2010 and RES 334/2012.

#### Accordance

All the methods were carried out in accordance with the approved guidelines.

## Additional Information

**How to cite this article**: Pérez-Alvarez, M. J. *et al.* Historical dimensions of population structure in a continuously distributed marine species: The case of the endemic Chilean dolphin. *Sci. Rep.* **6**, 35507; doi: 10.1038/srep35507 (2016).

## Supplementary Material

Supplementary Information

## Figures and Tables

**Table 1:** Haplotype frequencies (mtDNA) for the Chilean dolphin along its distribution range including the North and South areas.

Haplotypes	North	South	Total
H1	5	26	31
H2		3	3
H3	5		5
H4	6		6
H5	1		1
H6	3		3
H7		6	6
H8		1	1
H9		1	1
H10	2		2
H11		1	1
H12		1	1
H13	2		2
H14		1	1
Samples	24	40	64

**Table 2:** Genetic diversity (663 bp mtDNA control region) in the Chilean dolphin.

Area	mtDNA				
	n	Hap n	Hap r	*h* ± (SD)	π% ± (SD)
Total Area	64	14		0.75 ± 0.053	0.38 ± 0.002
North	24	7	6	0.86 ± 0.034	0.35 ± 0.002
South	40	8	6.3 ± 1.13	0.56 ± 0.086	0.24 ± 0.001

Number of samples (n), number of haplotypes (Hap n), number of haplotypes after rarefaction (Hap r), haplotype diversity (h), nucleotide diversity (π).

**Table 3:** Demographic scenarios, priors and posterior distributions used in Approximate Bayesian computation (ABC) analyses of the Chilean dolphin populations using populations using mitochondrial (mt) DNA and microsatellites.

Parameter name	Parameter abbreviation	Prior distribution mtDNA		Posterior distribution mtDNA	
		Type	Interval	Mean	95% CI
*Effective sizes*					
Population size	N	Uniform	100–100,000	8,000	4,700–9,900
Reduced population size	Nb	Uniform	5–1,000	480	281–967
*Time*					
Population expansion	t1	Uniform	5,000–24,000	9,790	5,250–18,600
Deglaciation starting time	t2	Uniform	10,000–25,000	15,100	10,200–24,000
Glaciation starting time	t3	Uniform	50,000–200,000	132,000	54,000–197,000
**Parameter name**	**Parameter abbreviation**	**Prior distribution Microsatelites**		**Posterior distribution Microsatelites**	
		**Type**	**Interval**	**Mean**	**95% CI**
*Effective sizes*					
Population size	N	Uniform	500–15,000	9,000	3,300–14,400
Reduced population size	Nb	Uniform	50–1,000	581	82–981
*Time*					
Population expansion	t1	Uniform	5,000–22,000	12,100	5,680–20,700
Deglaciation starting time	t2	Uniform	8,000–25,000	12,800	8,180–21,900
Glaciation starting time	t3	Uniform	50,000–200,000	128,000	54,300–196,000
