# Supplementary material for: Historical dimensions of population structure in a continuously distributed marine species: The case of the endemic Chilean dolphin
Source: Sci Rep. 2016 Oct 19;6:35507. doi: 10.1038/srep35507 (PMC5069719; doi:10.1038/srep35507)
Supplement: Supplementary Information [file srep35507-s1.pdf]

SUPPLEMENTARY INFORMATION

Historical dimensions of population structure in a continuously distributed marine species: The case of the endemic Chilean dolphin

*M.J. PÉREZ-ALVAREZ [1,2], C. OLAVARRÍA [2,3,4], R. MORAGA [2], C.S. BAKER [5], R.M. HAMNER [5,6], and E. POULIN [1]

[1] Instituto de Ecología y Biodiversidad (IEB), Facultad de Ciencias, Universidad de Chile, Las Palmeras 3425, Ñuñoa, Santiago, Chile

[2] Centro de Investigación Eutropia, Ahumada 131 Oficina 912, Santiago, Chile

[3] Fundación CEQUA, 21 de Mayo 1690, Punta Arenas, Chile.

[4] Centro de Estudios Avanzados en Zonas Áridas (CEAZA), Raúl Bitrán1305, La Serena, Chile

[5] Marine Mammal Institute and Department of Fisheries and Wildlife, Oregon State University, Hatfield Marine Science Center, 2030 SE Marine Science Drive, Newport, OR 97365, USA

[6]current address: Department of Life Sciences, Texas A&M University-Corpus Christi, 6300 Ocean Drive, Unit 5800, Corpus Christi, TX 78412-5800, USA

***Corresponding author:** María José Pérez-Alvarez, E-mail: mjose.perez@gmail.com, mjose.perez@eutropia.cl

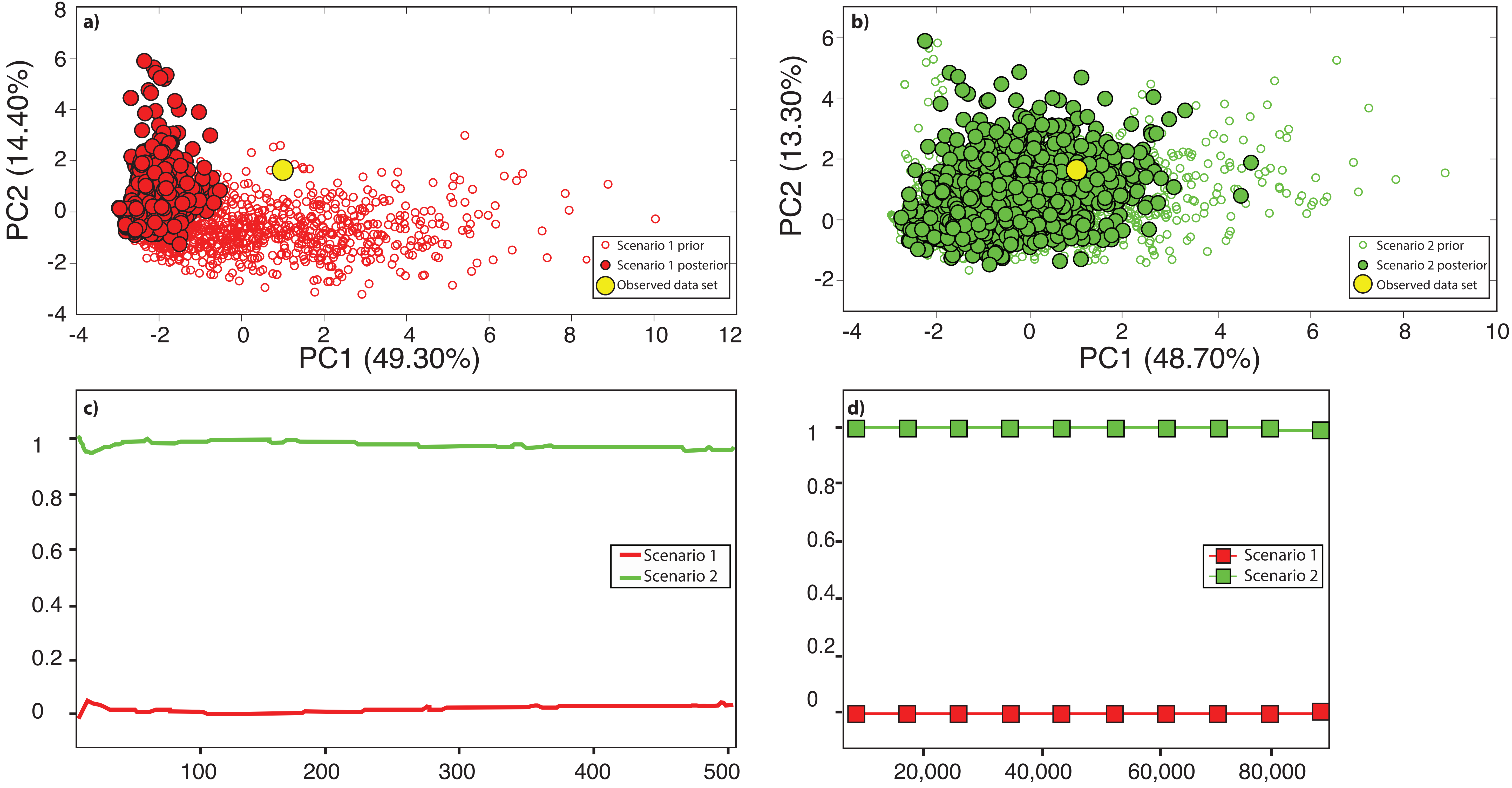

a)
PC2 (14.40%)
PC1 (49.30%)
Scenario 1 prior
Scenario 1 posterior
Observed data set
b)
PC2 (13.30%)
PC1 (48.70%)
Scenario 2 prior
Scenario 2 posterior
Observed data set
c)
Scenario 1
Scenario 2
d)
Scenario 1
Scenario 2

Supplementary Figure S1. Fine scale ABC analysis for the Chilean dolphin. Model checking (PCA) for the unselected scenario "In situ Refugia" (a) and the selected scenario "Northern Shift" (a). Direct estimation (a) and Logistic regression (d) showing support for the Northern Shift scenario.
